# The role of pulse timing in cardiac defibrillation

**DOI:** 10.3389/fnetp.2022.1007585

**Published:** 2023-01-04

**Authors:** Joshua Steyer, Thomas Lilienkamp, Stefan Luther, Ulrich Parlitz

**Affiliations:** ^1^ Max Planck Institute for Dynamics and Self-Organization, Göttingen, Germany; ^2^Institute for the Dynamics of Complex Systems, Georg-August-Universität Göttingen, Göttingen, Germany; ^3^ Institute of Biomedical Engineering, Karlsruhe Institute of Technology, Karlsruhe, Germany; ^4^ Faculty for Applied Mathematics, Physics, and General Science, Computational Physics for Life Science, Nuremberg Institute of Technology Georg Simon Ohm, Nürnberg, Germany; ^5^ German Center for Cardiovascular Research (DZHK), Partner Site Göttingen, Göttingen, Germany; ^6^ Institute of Pharmacology and Toxicology, University Medical Center Göttingen, Göttingen, Germany

**Keywords:** cardiac arrhythmias, defibrillation, electrophysiology, biophysical methods, dose-response curve, spiral waves, spatiotemporal chaos, nonlinear dynamics

## Abstract

Life-threatening cardiac arrhythmias require immediate defibrillation. For state-of-the-art shock treatments, a high field strength is required to achieve a sufficient success rate for terminating the complex spiral wave (rotor) dynamics underlying cardiac fibrillation. However, such high energy shocks have many adverse side effects due to the large electric currents applied. In this study, we show, using 2D simulations based on the Fenton-Karma model, that also pulses of relatively low energy may terminate the chaotic activity if applied at the right moment in time. In our simplified model for defibrillation, complex spiral waves are terminated by local perturbations corresponding to conductance heterogeneities acting as virtual electrodes in the presence of an external electric field. We demonstrate that time series of the success rate for low energy shocks exhibit pronounced peaks which correspond to short intervals in time during which perturbations aiming at terminating the chaotic fibrillation state are (much) more successful. Thus, the low energy shock regime, although yielding very low temporal average success rates, exhibits moments in time for which success rates are significantly higher than the average value shown in dose-response curves. This feature might be exploited in future defibrillation protocols for achieving high termination success rates with low or medium pulse energies.

## 1 Introduction

Sudden cardiac death, in many cases due to ventricular fibrillation (VF) Koplan and Stevenson (2009), causes around 700,000 deaths per year in Europe alone, and hence, remains one of the major public health issues Chugh (2017). In practice, such cardiac arrhythmias are most successfully terminated by applying external electric shocks to the heart, referred to as defibrillation. Demonstrated both experimentally Davy et al. (1987); Kwaku and Dillon (1996) and numerically Boegli et al. (2000); Bragard et al. (2013); Lilienkamp and Parlitz (2020), the dependence of the defibrillation success rate on the strength of the shock applied, which is called dose-response curve (DRC), is given by a sigmoid function. However, high energy shocks, being promising in terms of successful defibrillation, cause severe pain and tissue damage to the patient Holden et al. (2019). Another adverse side effect is that postshock arrythmias are much more probable for high defibrillation field strengths Cates et al. (1994); Fast and Cheek (2002). A major challenge in medical practice is thus, to minimize these tremendous side effects by significantly reducing the shock energy while keeping the probability for successful defibrillation high. Therefore, different types of approaches for energy reduction have been proposed, including a sequential application of low energy pulses Luther et al. (2011) or multi-stage electrotherapy Janardhan et al. (2014). In contrast, the current study points the way to a possible improvement in low-energy single-shock therapy. Using computational methods, we show that the success rate of external shocks may strongly fluctuate, a feature that might be exploited for the defibrillation of arrhythmic dynamics of the heart using low energy shocks. In our study, we used the Fenton-Karma model Fenton and Karma (1998), describing the electrical excitation dynamics of cardiac cells in a 2D computational domain and simulated chaotic spiral waves of cardiac excitation corresponding to fibrillation in a real heart. By means of the concept of virtual electrodes Trayanova et al. (1998); Pumir and Krinsky (1999); Bittihn et al. (2008), we then perturbed the spiral wave dynamics, aiming at terminating it using pulses of low energy. To conduct this study, we generated several trajectories of chaotic spiral wave dynamics and studied the time series of the success rate for terminating the complex dynamics by applying such perturbations. In this way, optimal times for the termination of cardiac arrhythmias were found. In contrast, the rather small success rate for low-energy shocks observed in the DRCs is a consequence of applying shocks at random times and averaging over these attempts. In terms of real-life defibrillation, this would mean that, if external low-energy shocks are applied at the right moment in time, defibrillation success rates might be significantly higher than when applying them at random times. By preventing the aforementioned negative side effects of high-energy defibrillation strengths, this could lead to a major improvement in medical application. The paper is structured as follows. In Section 2, we introduce the Fenton-Karma model, its implementation and how defibrillation is simulated. Section 3 reproduces the DRC qualitatively, which is then extended to a study of how the values of the success rate for fixed perturbation parameters are distributed temporally, i.e., within the time series. Afterwards, we perform a robustness study with respect to the fixed perturbation control parameters chosen herein and show how the waiting times for high success rates are distributed. Finally, in Section 4, we summarize the results obtained and critically discuss their generality and applicability to real-life defibrillation.

## 2 Methods

In this section, we will describe the model used for the study, as well as how it is implemented numerically. We will furthermore show how the complexity of chaotic cardiac dynamics (corresponding to fibrillation) is quantified in terms of phase singularities and explain how defibrillation is realized in our simulations.

### 2.1 The Fenton-Karma model and its numerical implementation

For the study of spatiotemporally chaotic spiral waves of cardiac excitation representing fibrillation, we choose a 2D implementation of the Fenton-Karma model Fenton and Karma (1998). Its dynamics is governed by the following reaction-diffusion equations describing the temporal evolution of the membrane potential *V*_m_ and the gating variables, *v* and *w*:

$$\frac{\partial V_{\text{m}}}{\partial t}=\nabla \cdot \tilde{D}\nabla V_{\text{m}}-\left[I_{\text{fi}}\left(V_{\text{m}},v\right)+I_{\text{so}}\left(V_{\text{m}}\right)+I_{\text{si}}\left(V_{\text{m}},w\right)\right]/C_{\text{m}},$$ (1)

$$\frac{\partial v}{\partial t}=\Theta \left(u_{\text{c}}-V_{\text{m}}\right)\left(1-v\right)\left[\frac{\Theta \left(V_{\text{m}}-u_{v}\right)}{\tau_{v1}^{-}}+\frac{\Theta \left(u_{v}-V_{\text{m}}\right)}{\tau_{v2}^{-}}\right]-\Theta \left(V_{\text{m}}-u_{\text{c}}\right)\frac{v}{\tau_{v}^{+}},$$ (2)

$$\frac{\partial w}{\partial t}=\Theta \left(u_{\text{c}}-V_{\text{m}}\right)\frac{1-w}{\tau_{w}^{-}}-\Theta \left(V_{\text{m}}-u_{\text{c}}\right)\frac{w}{\tau_{w}^{+}},$$ (3)

where $\tilde{D}$ denotes the diffusion tensor. For our work, we replaced the standard Heaviside functions Θ(*x*) by a continuous approximation in order to ensure differentiability Bittihn (2015):

$$\Theta \left(x\right)\to \frac{1}{2}\left(1+\tanh\left[k_{2}x\right]\right),$$ (4)

with the smoothing parameter chosen to be *k*_2_ = 10. The fast inward (fi), slow outward (so) and slow inward (si) ion currents appearing in Eq. 1 read:

$$I_{\text{fi}}\left(V_{\text{m}},v\right)=-\frac{v}{\tau_{d}}\Theta \left(V_{\text{m}}-u_{\text{c}}\right)\left(1-V_{\text{m}}\right)\left(V_{\text{m}}-u_{\text{c}}\right),$$ (5)

$$I_{\text{so}}\left(V_{\text{m}}\right)=\frac{V_{\text{m}}}{\tau_{\text{o}}}\Theta \left(u_{\text{c}}-V_{\text{m}}\right)+\frac{1}{\tau_{r}}\Theta \left(V_{\text{m}}-u_{\text{c}}\right),$$ (6)

$$I_{\text{si}}\left(V_{\text{m}},w\right)=-\frac{w}{2\tau_{\text{si}}}\left(1+\tanh\left[k\left(V_{\text{m}}-u_{\text{c}}^{\text{si}}\right)\right]\right).$$ (7)

Note that the Heaviside functions Θ occurring here are again approximated by the hyperbolic tangent function (Eq. 4). The parameter set used is given in Table 1.

**TABLE 1:** Fenton-Karma model parameter set used here Fenton et al. (2002).

	Value		Value
$\tau_{v}^{+}$	13.03 ms	*τ*_o_	12.5 ms
$\tau_{v1}^{-}$	19.6 ms	*τ*_r_	33.25 ms
$\tau_{v2}^{-}$	1,250 ms	*τ*_si_	29 ms
$\tau_{w}^{+}$	800 ms	*u*_c_	0.13 a.u.
$\tau_{w}^{-}$	40 ms	$u_{\text{c}}^{\text{si}}$	0.85 a.u.
*τ*_d_	0.45 ms	*u_v_*	0.04 a.u.
*C*_m_	1 a.u.	*k*	500

We chose a 2-dimensional computational domain, D , with *N*_*x*_ × *N*_*y*_ = 100 × 100 grid points and an equidistant spacing of *h*_*x*_ = *h*_*y*_ = 1 mm. Furthermore, we assumed a constant, homogeneous and isotropic diffusion given by $\tilde{D}=D=0.2{mm}^{2}/ms$ , which reduces the first term of Eq. 1 to the Laplace operator Δ acting on the membrane potential *V*_m_. The Laplace operator was discretized *via* a finite differences nine-point stencil Bittihn (2015), while the time-wise discretization was realized with the explicit Euler algorithm Press et al. (2007), using an integration time stepping of d*t* = 0.2 ms. As boundary condition for the computational domain D , we chose no-flux boundary conditions Bittihn (2015) which were realized by extending ∂D with ghost points. For generating the spiral wave dynamics investigated, we used a modification of the cross pacing protocol, as described in the Supplementary Material. In order to have a measure for whether a state is governed by (chaotic) spiral waves, we evaluated the phase singularities on D Iyer and Gray (2001):

$$n^{\text{top}}={\frac{1}{2\pi }\oint }_{C}\nabla \theta \;\text{d}l\;,$$ (8)

where C is a closed integration path along eight grid points around each point of reference and *θ* is the phase characterizing the dynamics at each grid point Datseris and Parlitz (2022). A more detailed description of the phase, its calculation as well as the parametrization of the integration path is given in the Supplementary Material. Phase singularities are associated with the organizing centers of the spiral wave dynamics of cardiac excitation. Equation 8 yields *n* ^top^ = 0 if there is no phase singularity enclosed by C and *n* ^top^ = ± 1 if there is one enclosed. If at least one phase singularity is detected, we consider the dynamics to be dominated by spiral waves of excitation and thus, to represent arrhythmic dynamics of the heart.

We now want to briefly introduce the concept of virtual electrodes which will be used to simulate the defibrillation process. The heart exhibits heterogeneities of different sizes and configurations, caused, e.g., by blood vessels, discontinuities between sheets of fibers and bundles, or collagen existent in the extracellular space, resulting in a change in tissue conductivity Trayanova et al. (1998). When applying an external electrical field for a sufficiently long time, ions in the tissue are exposed to a Lorentz force which, in turn, shifts them such that they assemble at regions of differing conductivities. If the resulting hyper- and depolarization exceeds a certain threshold, an action potential is triggered at the heterogeneities, resulting in excitation waves Pumir and Krinsky (1999); Bittihn et al. (2008); Lilienkamp (2018). In this sense, in the presence of an electric field, the inhomogeneities act as virtual electrodes. The activation threshold of virtual electrodes depends on the shape (curvature) and size Bittihn et al. (2008); Bezekci et al. (2015) of the heterogeneities and the higher the external field strength the more heterogeneities emit a local excitation wave. This relationship between field strength and the number of activated virtual electrodes is exploited in the current study, where the number of local perturbation sites *N*_pert_ is used as a control parameter representing the applied shock energy.

The perturbation sites (representing heterogeneities) were chosen with a fixed size of 2 × 2 grid points and randomly distributed in space without overlap. The effect of the external electric field is approximated by an instantaneous increase of the membrane potential at the perturbation sites

$${\left(V_{\text{m},i}^{\text{pert}}\right)}^{2\times 2}={\left(V_{\text{m},i}^{\text{curr}}\right)}^{2\times 2}+{\left(\Delta V_{\text{m},i}\right)}^{2\times 2}\;\;\;\forall \;i\in \left\{1,\ldots ,N_{\text{pert}}\right\}$$ (9)

with an amplitude of ${\left(\Delta V_{\text{m}}\right)}^{2\times 2}=0.5$ a.u. The number of perturbation sites *N*_pert_ approximately corresponds to the fixed external field strength applied to the cardiac tissue. Finally, as a measure for whether the spiral wave dynamics was successfully terminated, i.e., whether the defibrillation attempt was successful, we checked whether 500 ms after the perturbation (no) phase singularities still existed in the computational domain by evaluating Eq. 8. The absence of phase singularities necessarily leads to convergence of the system towards the steady state, i.e., the electrical excitation dies out on D , such that the regular sinus rhythm could be restored by the sinus node (which is, however, not part of the current model).

## 3 Results

In what follows, we will summarize our results. First of all, we reproduced the typical sigmoid shape of the dose-response curve for the *in silico* defibrillation attempts. We then analyzed time series of the termination success rates with a focus on their peak structure, followed by a robustness study of the rather specific choice of perturbation parameters made here.

### 3.1 Distribution of defibrillation success rates for fixed shock doses

**FIGURE 1:**
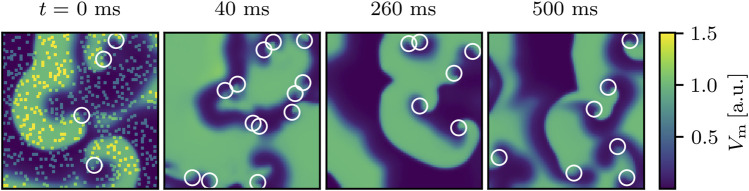
An example for an unsuccessful termination attempt for chaotic cardiac excitation using *N*_pert_ = 500 randomly distributed perturbation sites (visible as yellow dots immediately after the shock at *t* = 0 ms). White circles indicate phase singularities. At *t* = 0 ms the phase singularities were computed immediately before the perturbation. After 500 ms, the state still exhibits phase singularities and since they are considered to be the organizing centers of the spiral wave dynamics, they clearly indicate that the termination attempt failed.

**FIGURE 2:**
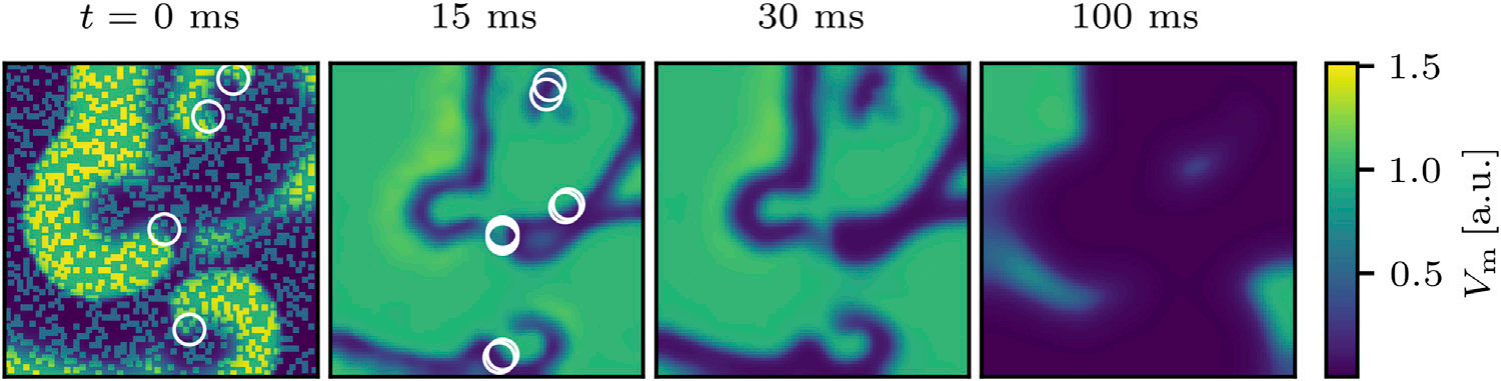
Successful termination of spiral wave dynamics of cardiac excitation by applying *N*_pert_ = 1,000 local stimuli to the same state as shown in Figure 1. White circles indicate phase singularities and were computed for *t* = 0 ms before the perturbation was applied. Even though the number of phase singularities increases shortly after applying the perturbations (not shown here), they died out already 30 ms after applying the perturbations and the remaining excitation converges quickly to the steady state as shown exemplary at *t* = 100 ms.

First, we compute the dose-response curve (DRC), describing the dependence of the success rate on the amount of perturbation sites *N*_pert_ used to terminate the chaotic excitation dynamics and show that the success rate values are broadly distributed around their mean values. Note that, by making use of the concept of virtual electrodes, *N*_pert_ is here considered to directly correlate with the electric field strength applied to the arrhythmic heart. The defibrillation and the determination of whether the attempt was successful was realized as described in Section 2. As a start, we show an example for an unsuccessful perturbation attempt and an example for a successful one (defibrillation). The failed attempt using *N*_pert_ = 500 is shown in Figure 1, which also illustrates that the size of the spatial domain is larger than twice the typical diameter of the spiral waves. If, however, not a single phase singularity is detected anymore on D after 500 ms, the perturbation is considered to be successful like in the example shown in Figure 2 where phase singularities are already absent 30 ms after applying the shock at, in this case, *N*_pert_ = 1,000 perturbation sites. In general, a very large number of perturbation sites corresponds to a high shock strength applied to the heart which depolarizes the whole tissue. As a consequence, it is impossible for the spiral wave dynamics to further evolve due to the refractory period not leaving any space for them to propagate.

**FIGURE 3:**
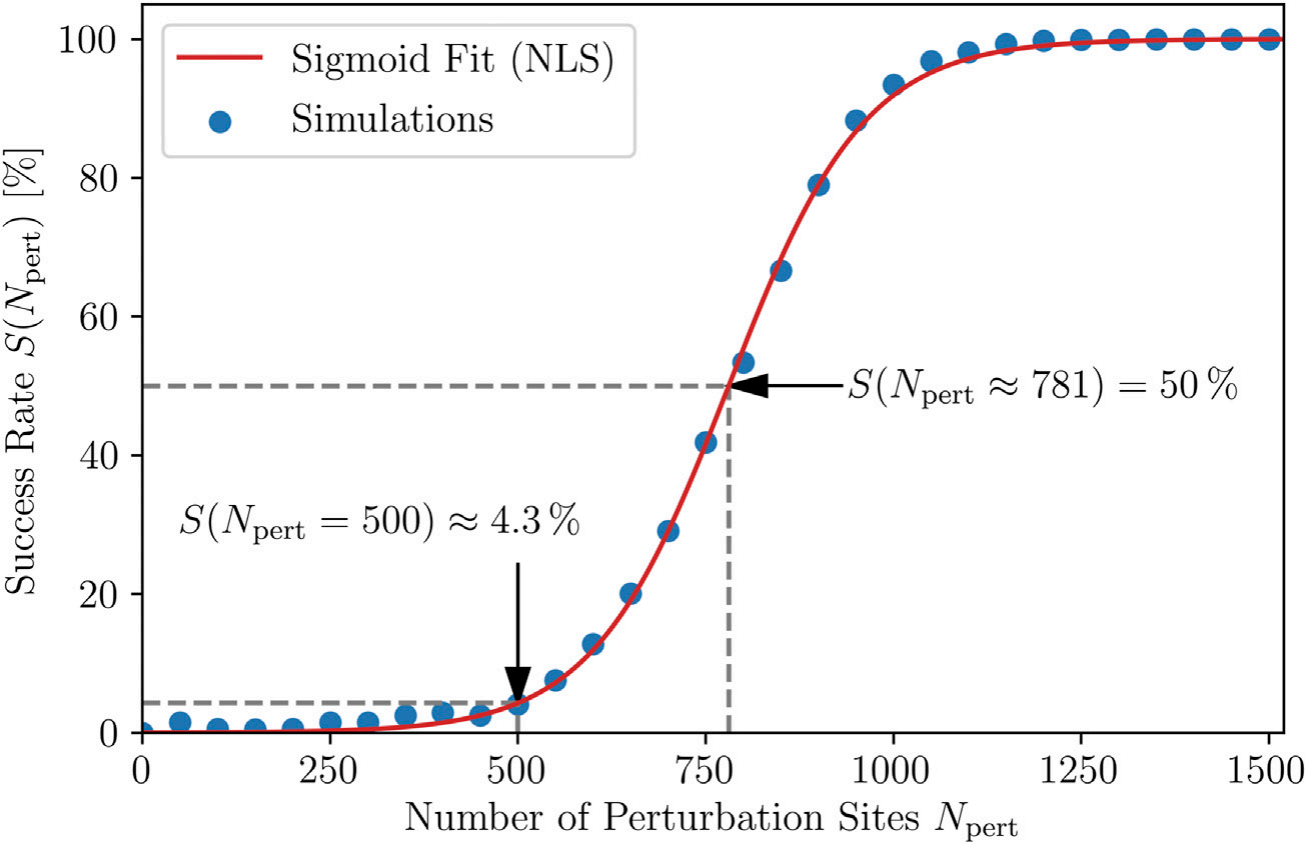
Dose-response curve. The dependence of the probability *S* of a successful termination of the chaotic dynamics on the number of perturbation sites, *N*_pert_, corresponding to the strength of the applied shocks. Each point corresponds to 20 × 50 = 1,000 simulations. The simoid fit *S* = 100/(1 + *c* exp (−*kN*_pert_)) is obtained using nonlinear least squares (NLS) to determine the parameters *c* = 5,632 and *k* = 0.01106. The inflection point, as well as the average success rate of 4.3 % for the case of using *N*_pert_ = 500 perturbation sites are indicated, the latter being referred to in the rest of this study.

For the DRC, we used 30 different fixed numbers of perturbation sites, *N*_pert_ ∈ {50, 100, 150, … , 1500}. For each of these fixed numbers, we randomly generated 50 different spatial configurations and applied them independently to 20 different chaotic states (CS). Thus, for the DRC, we have a total amount of

$$\left(\text{no. of CS}\right)\times \left(\text{no. of }N_{\text{pert}}\right)\times \left(\text{no. of conf.}\right)=20\times 30\times 50=3\times 10^{4}$$

termination attempts. The corresponding relation between the success rate and the number of perturbation sites applied, *S*(*N*_pert_), is shown in Figure 3. The shape is indeed sigmoidal and thus qualitatively reproduces the DRCs from experimental Davy et al. (1987); Kwaku and Dillon (1996) and numerical Boegli et al. (2000); Bragard et al. (2013); Lilienkamp and Parlitz (2020) studies.

**FIGURE 4:**
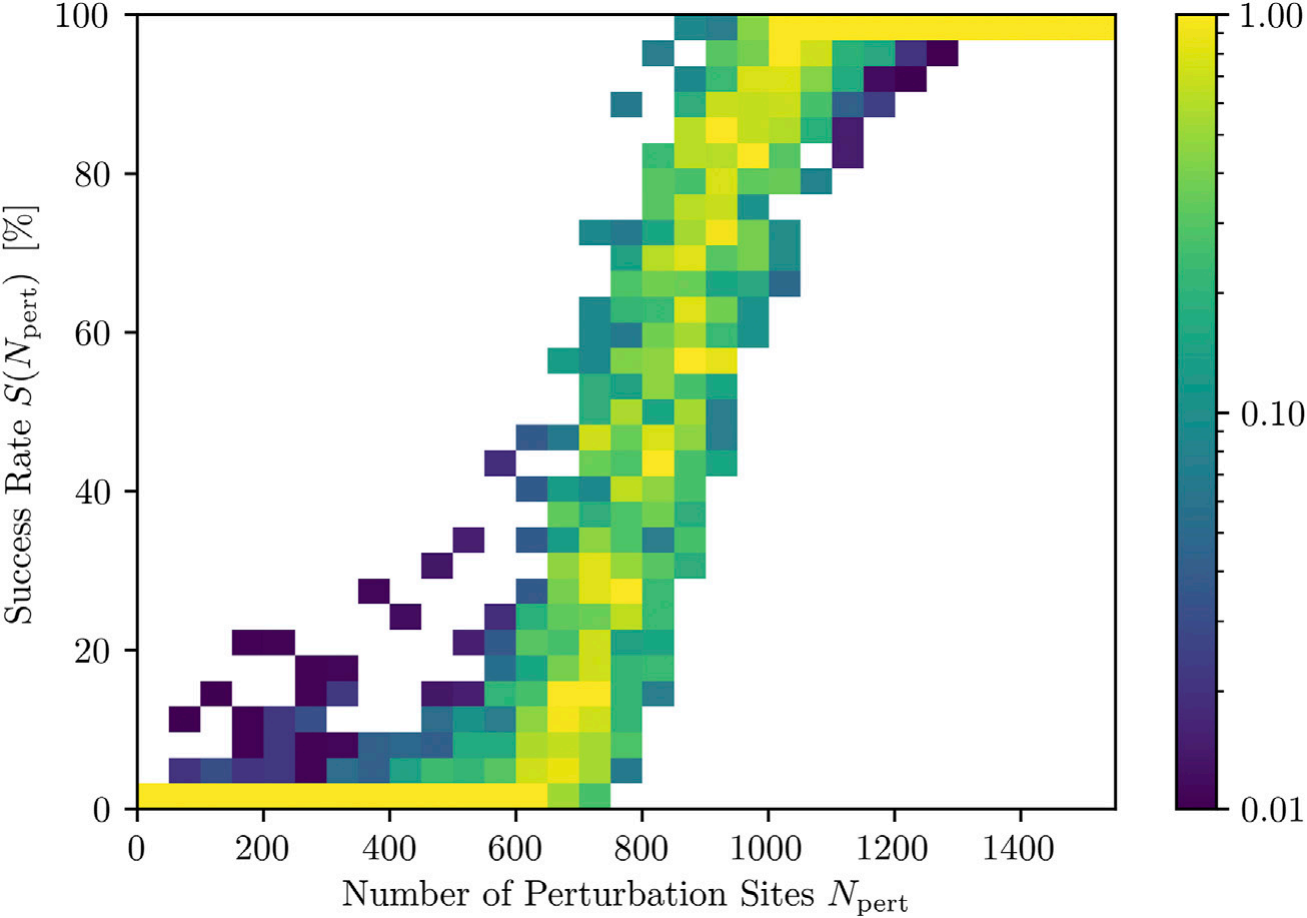
Distributions of success rates (color coded) for different numbers of activated perturbation sites, *N*_pert_. Note that the colorbar, indicating the occurrence of a success rate range, is chosen logarithmically and normalized to the most frequently occurring success rate range per *N*_pert_.

Since, due to the averaging over each termination attempt, the representation of the DRC in Figure 3 gives only limited insight into the actual values for the success rate occurring for each fixed *N*_pert_, we also investigated the distribution of *S*(*N*_pert_) for all fixed *N*_pert_ for the same spacing. The resulting success rate distribution DRC is given in Figure 4. It is composed of ten times more defibrillation attempts compared to the averaged DRC shown in Figure 3:

$$\left(\text{no. of CS}\right)\times \left(\text{no. of }N_{\text{pert}}\right)\times \left(\text{no. of conf.}\right)=100\times 30\times 100=3\times 10^{5}.$$

As one can see, perturbations with very low or very high *N*_pert_ result in narrow distributions of success rate values. Within intermediate ranges of *N*_pert_, on the other hand, especially around the inflection point of the DRC, we have a large range of success rates *S*(*N*_pert_). This, in turn, implies that the success of a termination attempt is not only dependent on the number of perturbation sites, *N*_pert_, but also on the state (point in time) to which the stimuli are applied.

### 3.2 Probability of defibrillation success strongly depends on time when shock is applied

We now want to show how the success rate is time-dependent or, more specifically, that its time series shows rather high maxima which are not just small deviations around the mean success rate. We will then show that these peaks have a non-vanishing width. As already mentioned in the introduction, the aim of this study is to terminate arrhythmic cardiac excitation waves with perturbation parameters corresponding to low defibrillation field strengths, thus causing less pain and tissue damage due to the shocks, while keeping the termination success rate significantly higher than on average for the same energy. For the perturbations, we chose a fixed number of *N*_pert_ = 500 perturbation sites and a fixed amplitude of $\Delta V_{\text{m}}^{2\times 2}=0.5$ a.u. For this choice, the averaged DRC, Figure 3, yields a very low success rate of *S*(*N*_pert_ = 500) ≈ 4.3 % which corresponds to the success rate of applying shocks at random times (states) to the chaotic cardiac excitation. This is an unacceptably small value for clinical application.

Given the success rate distribution DRC, Figure 4, we analyze success rate time series *S* (*t*_appl_) in terms of a whether they exhibit a significant peak structure. Compared to both DRCs shown in Figures 3, 4, we strongly increased the amount of realizations for the success rate analyses. First of all, we generated 100 realizations of the time evolution (i.e. 100 trajectories with different initial conditions), each being 10 s long, exhibiting chaotic spiral wave dynamics which were then analyzed time-wise in terms of their response to applied perturbations. The 100 chaotic trajectories were each sampled with a rate of 100.1 Hz, resulting in sequences of states. Each of these states was then perturbed individually 100 times using 100 different, randomly generated fixed spatial distributions of 500 perturbations sites (acting like virtual electrodes). In this way, a success rate was estimated for each of the 1,001 states (or points in time separated by d*t* = 10 ms) along the chaotic trajectory (CT) respresenting a fibrillation episode. The total amount of termination attempts realized is thus:

$$\left(\text{no. of CT}\right)\times \left(\text{no. of }t_{\text{appl}}\right)\times \left(\text{no of conf.}\right)=100\times 1001\times 100\approx 10^{7}.$$

**FIGURE 5:**
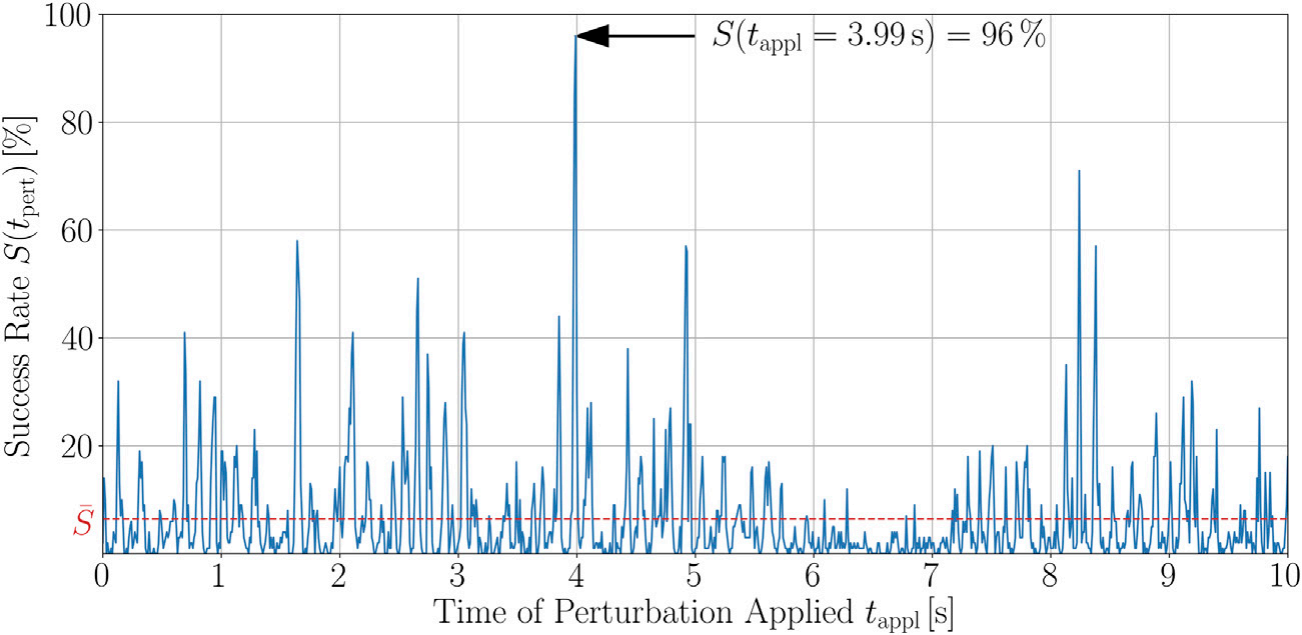
Time series of the success rate, *S*(*t*_appl_), for perturbing a trajectory of chaotic cardiac excitation. With a temporal resolution of d*t*_appl_ = 10 ms, the states along the trajectory are perturbed using 100 different spatial configurations of *N*_pert_ = 500 perturbation sites that were randomly chosen before starting the analysis and kept fixed along the trajectory. For each of the 100 defibrillation attempts, the success rate was evaluated according to Section 2 and the spiral wave dynamics termination success was estimated in terms of whether phase singularities still exist 500 ms after the perturbation. The temporal average of the success rate over the whole trajectory is $\bar{S}\approx 6.5\;\%$ . The peaks have a certain width and are not just particularly favorable points in time for shocking, as shown exemplary for a higher resolved peak in Figure 6.

First, we want to discuss the structure of the time series and the resulting implications. A typical example of such a time series of the success rate is given in Figure 5. As can be clearly seen, there is a well-established peak structure of the success rate and thus, a time-dependence of it. This means that there are short time intervals within which the termination of chaotic excitation waves is much more probable than on average. As a matter of fact, if external stimuli are applied to the most favorable state of the time series shown in Figure 5, which is at *t*_appl_ = 3.99 s, the probability to successfully terminate the unwanted dynamics is almost fifteen times higher than when averaging over all success rates of the 1,001 different states the trajectory consists of. On average, such a success rate would be reached only with twice as much perturbations, see Figure 3. The temporally averaged success rate for the trajectory shown in Figure 5 is $\bar{S}\approx 6.5$ % which agrees well with the order of value of the averaged DRC, Figure 3. The apparent difference (6.5 % vs. 4.3 %) is due to the fact that in the DRC, for each number of perturbation sites, 20 × 50 = 1,000 realizations are evaluated, whilst the time series shown in Figure 5 is composed of more than 100,000 termination attempts. Note that, for a sufficient number of realizations (perturbed states), the average termination success rate of a trajectory, $\bar{S}$ in Figure 5, and of randomly chosen single chaotic states, as for the averaged DRC, Figure 3, may be considered equivalent quantities and correspond to the termination success probability of defibrillating at random times.

**FIGURE 6:**
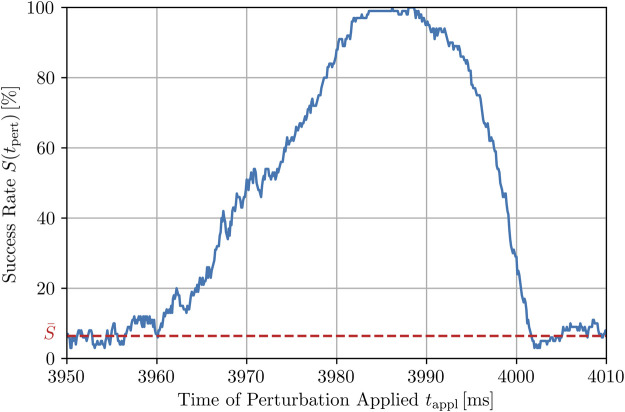
Higher-resolution plot of the peak highlighted in Figure 5 at 3.99 s for *N*_pert_ = 500, using smaller time steps for the application of local stimuli, d*t*_appl_ = 0.1 ms. The full width at half maximum (FWHM) is roughly 25 ms. However, the whole peak and thus, the time window with a success rate being (in parts significantly) higher than the average success rate $\bar{S}$ has a width of about 40 ms.

For all of the 100 trajectories, we found qualitatively similar success rate time series. Altogether, the main message of such peak structures for *S*(*t*_appl_) is that, if perturbations are efficiently applied, i.e., at an appropriate time, one can yield success rates being, in parts significantly, higher than the temporal average, $\bar{S}$ , over the trajectory. Another remarkable observation of the exemplary realization shown in Figure 5 (and others not shown here) is that the peaks possess a certain width. In order to illustrate this feature, we generated higher resolution plots of several of the peaks from the chaotic trajectories (i.e., again with *N*_pert_ = 500), using a time stepping of d*t*_appl_ = 0.1 ms (which requires the initial temporal integration step to be halved) for the termination attempts yielding a resolution being 10^2^ times higher than used otherwise within the study. In Figure 6, we can exemplarily see a higher resolution of the largest peak of the time series indicated in Figure 5, i.e., at *S*(*t*_appl_ = 3.99 s) = 96 % which has a width of about 40 ms with respect to the mean value of the success rate $\bar{S}$ . A similar width was also observed for other high-resolution success peaks (not shown here). The fact that high success rates do not occur within extremely narrow peaks but in relatively broad time windows is important with regard to their possible future use in practical experimental or clinical applications.

### 3.3 The peak structure is sufficiently robust to variations of perturbation parameters

**FIGURE 7:**
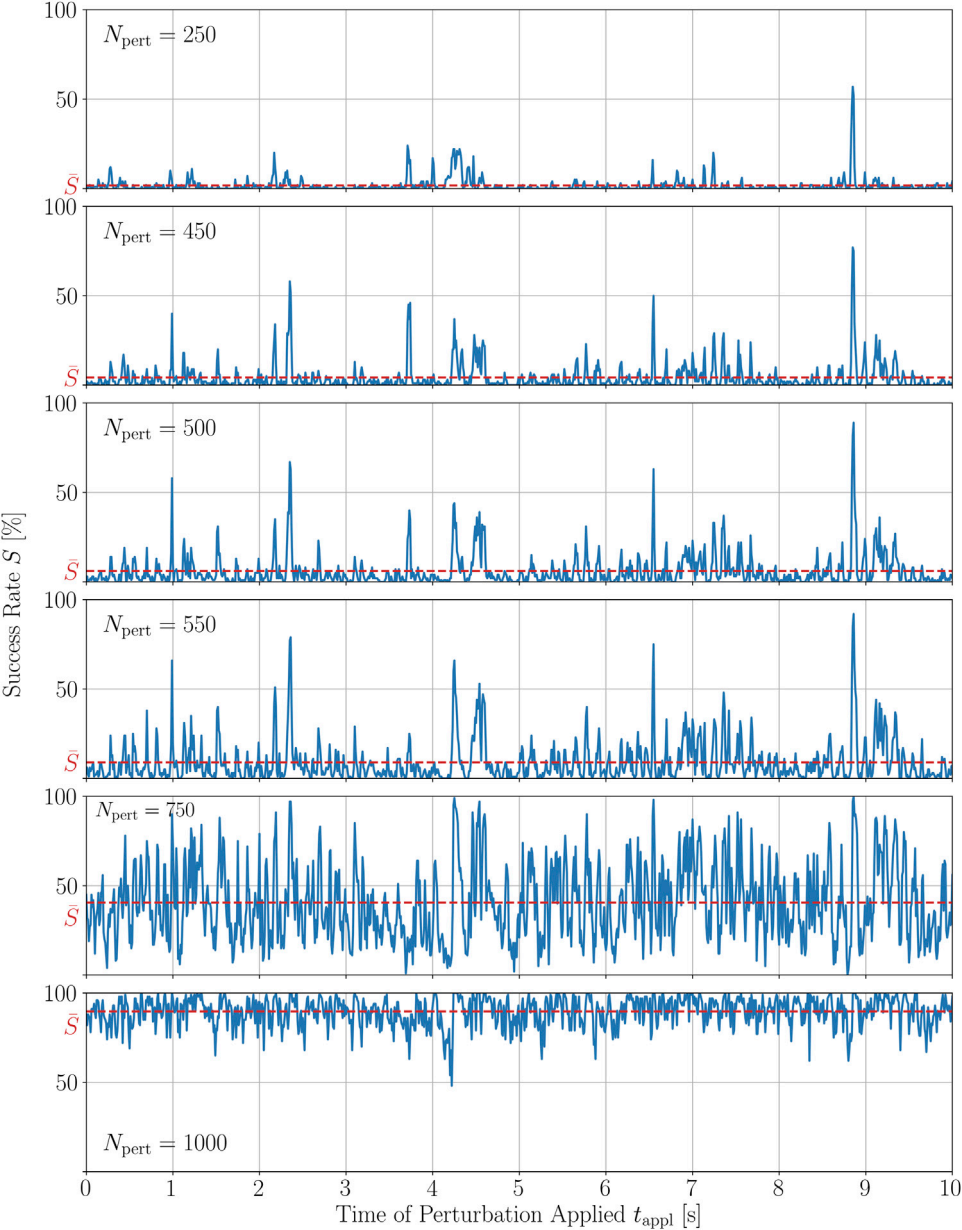
Time series of the termination success rate of a single trajectory of chaotic cardiac excitation for different amounts of local stimuli applied *N*_pert_ to the system. $\bar{S}$ represents the corresponding average success rate of the time series *S*(*t*). Perturbations are applied as described in the caption of Figure 5.

Since the choices of the perturbation parameters, *N*_pert_ = 500 and $\Delta V_{\text{m}}^{2\times 2}=0.5$ a.u., are rather specific, in what follows, we will show that *S*(*t*_appl_) is sufficiently robust to changes in these parameters, starting with an investigation of the robustness to changes in the number of local stimuli added to the system, *N*_pert_. In order to do so, we exemplarily show a success rate time series of one out of the 100 trajectories perturbed with different *N*_pert_ ∈ {250, 450, 500, 550, 750, 1000}, respectively, evaluating again 100 different configurations of spatial distributions of perturbation sites for the same states, *t*_appl_. In Figure 7, one can see the resulting time series of the success rate *S*(*t*_appl_) if the very same trajectory is perturbed with these different numbers of perturbation sites. As *N*_pert_ is increased, the peak structure is, at least partially, preserved. Furthermore, most peaks that are preserved when applying more perturbations, grow in height. A good example where this can be observed is the highest peak for *N*_pert_ = 500 at around *t*_appl_ = 8.86 s. It does not only dominate the time series for *N*_pert_ = 500 but also the ones for the other numbers of perturbation sites used, growing in height the more *N*_pert_ are used. Only for the time series using 750 and 1,000 perturbation sites, the phenomenon of a few dominating peaks cannot be observed anymore because most of their peaks are generally very high. In terms of the hierarchy of peak heights, however, there does not seem to be a definite rule. The three peaks for *N*_pert_ = 250 appearing after 2 s have a different order in terms of their height if defibrillation attempts with more local stimuli are evaluated, for example. Finally, there are also examples where peaks present for a small number of perturbation sites seem to disappear as *N*_pert_ is increased. An example for this can be observed for the peak at 4 s for the time series with *N*_pert_ = 250 which disappears for *N*_pert_ ∈ {450, 500, 550} before finally recovering slowly as more perturbations sites are applied. In general, however, the corresponding temporal averages, $\bar{S}$ , agree well with the averaged DRC in Figure 3, as it can be seen by taking a look at the monotonically increasing $\bar{S}$ as *N*_pert_ is increased.

In order to quantify these observations, we calculated the Spearman and Pearson correlation coefficients, *r*_s_ and *ρ*_p_, respectively, between all combinations of time series given in Figure 7. The Spearman correlation is a measure for how well a monotonic function can approximate the relationship between two samples or, in this case, between two time series. In particular, the Spearman coefficients between *S*(*t*_appl_) for *N*_pert_ = 500 and *S*(*t*_appl_) of the closest numbers investigated here, i.e., 450 and 550, are 0.78 and 0.85, respectively. The complete set of Spearman and Pearson correlation coefficients between the success time series for different numbers of perturbation sites is given in the Supplementary Material. The Pearson correlation coefficient measures the linear dependence of two variables. For small changes in *N*_pert_ = 500, so again *N*_pert_ = 450 and *N*_pert_ = 550, the Pearson correlation coefficient of the corresponding time series *S*(*t*_appl_) is even higher, *ρ*_p_ = 0.93 in both cases, respectively. The relatively large value of the Spearman coefficient and the even larger value for the Pearson coefficient between time series perturbed with similar numbers of perturbation sites, agrees well with the qualitative observation that, most of the peaks are preserved with growing heights, whereas some of them seem to vanish as *N*_pert_ is increased. Hence, we may conclude that the peak structure is sufficiently robust, especially to small variations in *N*_pert_.

**FIGURE 8:**
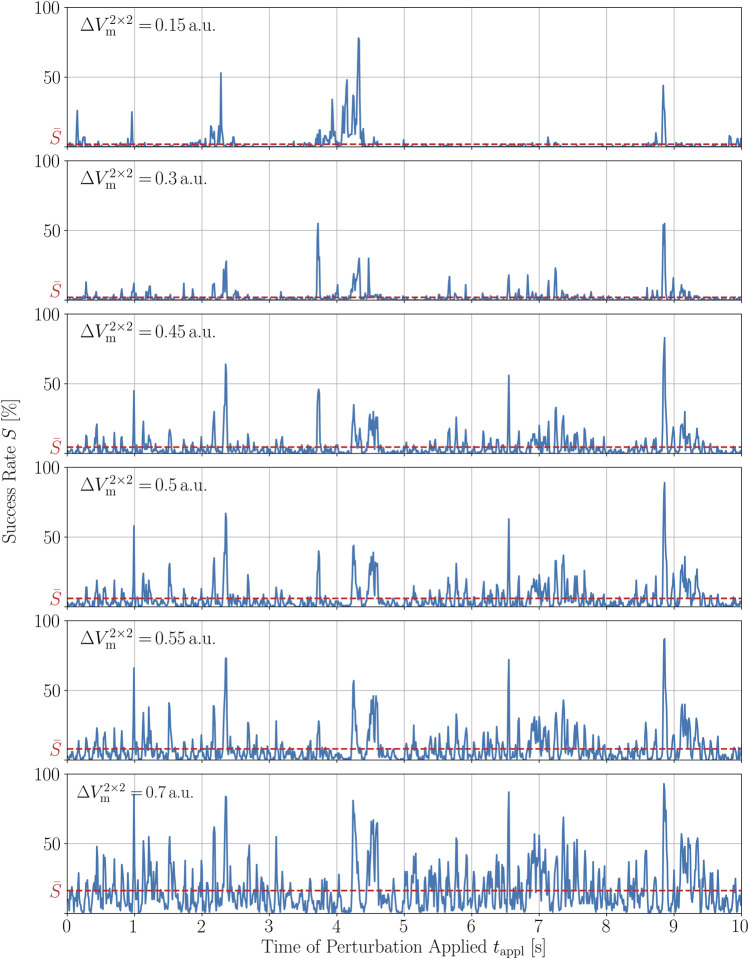
Time series of the termination success rate of a single chaotic trajectory for different magnitudes of the perturbations, $\Delta V_{\text{m}}^{2\times 2}$ , applied to the system. $\bar{S}$ represents the average success rate of the respective time series.

We now want to show a similar robustness of *S*(*t*_appl_) to changes in the second perturbation parameter, the amplitude of local stimuli, $\Delta V_{\text{m}}^{2\times 2}$ . Therefore, we repeated the previous investigation, this time by varying the magnitude of the perturbations and not their number, using $\Delta V_{\text{m}}^{2\times 2}\in \left\{0.15,0.3,0.45,0.5,0.55,0.7\right\}$ a.u. In principle, the situation is similar to the time series with varying *N*_pert_, i.e., rather large parts of the peak structure is preserved if the perturbation amplitude is increased, as illustrated in Figure 8. However, for very small amplitudes, $\Delta V_{\text{m}}^{2\times 2}=0.15$ a.u., this does not apply. Taking a look at the peaks of this success rate time series and compare it with the peaks of *S*(*t*_appl_) with a perturbation magnitude twice as large, we see that most of the peaks are attenuated which is counter-intuitive to the observations made so far. When taking a look at the correlation coefficients between the *S*(*t*_appl_) perturbed with the magnitude used here, $\Delta V_{\text{m}}^{2\times 2}=0.5$ a.u., with its two neighboring magnitude values investigated, $\Delta V_{\text{m}}^{2\times 2}=0.45$ a.u. and $\Delta V_{\text{m}}^{2\times 2}=0.55$ a.u., however, we obtain values similar to the variation of *N*_pert_ around 500 perturbations. The Spearman correlation coefficients are *r*_s_ = 0.8 and *r*_s_ = 0.87, respectively, whereas the Pearson correlation coefficient is the same for varying $\Delta V_{\text{m}}^{2\times 2}=0.5$ a.u. in both directions, namely *ρ*_p_ = 0.95. The complete set of correlation coefficients between all trajectories with varying perturbation magnitude is given in the Supplementary Material as well. This demonstrates that also variations of the perturbation amplitude chosen for this study are sufficiently robust in terms of the success rate time series. The fact that the peak structure is not completely preserved when changing *N*_pert_ and $\Delta V_{\text{m}}^{2\times 2}$ is very likely a consequence of the chaotic nature of the trajectory and hence, probably inevitable. However, the general finding here is that the peak structure is relatively robust to changes in the perturbation parameters.

### 3.4 Relation between peak height and waiting times

Even though most trajectories do exhibit states with relatively large success rates, these peaks do not occur very frequently, as illustrated by the time series shown so far. We therefore show here how waiting times for peaks increase dramatically the larger we require a peak to be and thus, the success rate for defibrillation. For each state of a trajectory investigated, given by its time of occurrence *t* ^*i*^, the waiting time is defined as the distance in time from this state to its closest temporally forward peak of the required height *h*

$$t_{\text{wait}}^{i}=t_{h}^{i}-t^{i}.$$ (10)

**FIGURE 9:**
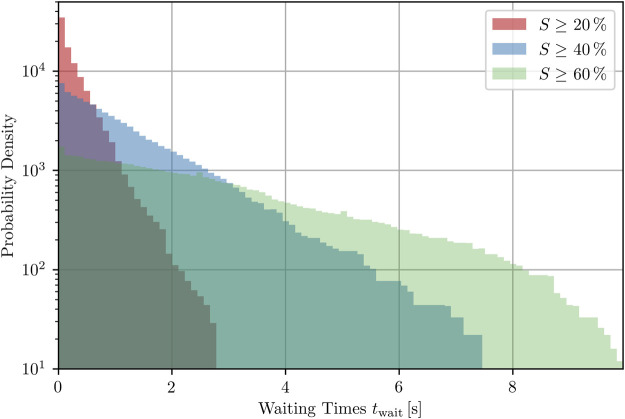
Probability density of waiting times for the next peak (with specific minimal heights, respectively) to occur inside one trajectory, averaged over all states of 100 trajectories that do not correspond to a peak of the success rate, see Eq. 10.

Note that, if *t* ^*i*^ itself meets the condition (i.e., is the point in time at which a peak of given height *h* occurs), then $t_{\text{wait}}^{i}=0$ ms. Furthermore, states after the last $t_{h}^{i}$ of a specific trajectory are not considered as there is no subsequent closest temporally forward peak (state) for them to refer to. In Figure 9, the distribution of the waiting times of each sampled state the 100 trajectories are composed of is plotted semi-logarithmically for *h* ∈ {20, 40, 60} %. The waiting times for a peak with a termination success rate of 20 % are usually not longer than 2 s and the fraction of waiting times smaller than 1 s is 0.94. For 60 %, however, this fraction reduces dramatically down to 0.26. In the context of defibrillation, this means that one usually does not have to wait long for a time interval inside which the success rate is multiple times larger than on average, but the success rates are the lower the less one wants to wait for the defibrillation to be applied, on average.

**FIGURE 10:**
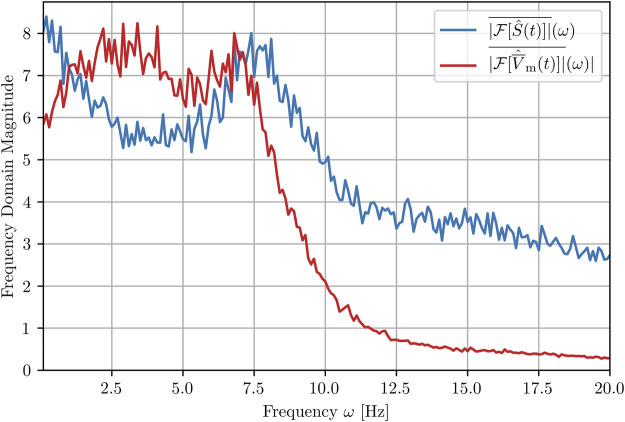
Mean amplitude spectra of the success rate and the membrane voltage averaged over 100 realizations (trajectories). Note that the hat denotes the normalization of both quantities before the carrying out the FFT in order to obtain comparable magnitudes and does not itself denote an FFT.

Finally, we show here how the peak structure, *S*(*t*_appl_), is characterized in terms of its frequency by comparing the frequency spectra obtained *via* Fourier transformation (FFT) of both, the success rate and the mean of the membrane potential time series, S(t_appl_) and ${\bar{V}}_{\text{m}}\left(t\right)$ where, for simplicity, *t*_appl_ = *t*. In order to do so, we normalized each of the 100 time series, *S*(*t*) and ${\bar{V}}_{\text{m}}\left(t\right)$ , with respect to their corresponding maximal value, denoted by $\hat{S}\left(t\right)$ and V^¯m(t) (not to be confused with the hat notation for Fourier transforms). We then calculated the FFT of these time series, evaluated their modulus and finally averaged over the moduli of the FFTs associated with each trajectory, yielding the amplitude spectra of both quantities. The comparison of these frequency spectra of ${\bar{V}}_{\text{m}}\left(t\right)$ and *S*(*t*) is shown in Figure 10.

The strongest frequency components of the 100 time series ${\bar{V}}_{\text{m}}\left(t\right)$ occur between 2 and 7 Hz. This shape of the spectrum makes it difficult to specify a single frequency as “the” dominant frequency, but the range [2 Hz, 7 Hz] agrees with experimental data on ventricular fibrillation in human hearts, for example in Pandit and Jalife (2013). If a dominant frequency can be clearly identified, its inverse corresponds to the (average) rotation period of the spiral waves Mandapati et al. (1998) and thus provides a relevant time scale characterizing the chaotic wave dynamics. An interesting observation is that the peak of the success rate spectrum at 7.5 Hz occurs at the upper limit of the spectrum of the membrane voltage. This means that within each rotational period of even the fastest spiral waves (on average), there is a state for which the application of perturbation would be particularly successful in terminating the spatiotemporal chaos.

## 4 Discussion and conclusion

Defibrillation shocks used to terminate the complex electrical excitation dynamics in the heart during ventricular fibrillation must be of high energy to achieve a high success rate in terminating the arrhythmia, resulting in pain and damage to heart tissue Holden et al. (2019). In practice, these shocks are applied at random times during the fibrillation. Therefore, in this study, we addressed the question of whether there are more favorable times (or states) in the temporal evolution of an arrhythmia at which even medium- or low-energy shocks can terminate the chaotic state corresponding to fibrillation with relatively high probablility. To investigate this question, we performed extensive numerical simulations using the Fenton-Karma model and an implementation of multi-site pacing by virtual electrodes. With these simulations, we were not only able to reproduce the characteristic sigmoidal shape of the dose-response curve, but also found strong fluctuations of the termination success rate during the time evolution of the complex dynamics of cardiac activity resulting in well-established peak structures of success rate time series. In these simulations, it was found that even relatively low energy shocks could terminate the chaotic dynamics if they were applied in short time windows in which the termination success was particularly high. If such fluctuations of termination success could be predicted, they might be exploited in future defibrillation protocols for terminating arrhythmias with low or medium pulse energies.

Since the underlying model represents a simple model for cardiac excitation dynamics, the simulations made here to study success rate time series of trajectories representing arrhythmias only qualitatively describe the dynamics of a real heart. First of all, the Fenton-Karma model is of low to moderate complexity and thus, may not incorporate cardiac dynamics with all desired details. Furthermore, out of several parameter sets suggested for the Fenton-Karma model, we only used a specific one. Thus, the results found in the study presented here need to be confirmed in future investigations. As a starting point, one could repeat the simulations using different parameter sets of the Fenton-Karma model Fenton et al. (2002), corresponding to different properties of cardiac dynamics. Besides being computationally more expensive, the results presented should also be verified using more detailed (ionic) models of cardiac excitation, such as the 4-variable Bueno-Orovio-Cherry-Fenton model Bueno-Orovio et al. (2008), the 8-variable Beeler-Reuter model Beeler and Reuter (1977) or the 17-variable Ten Tusscher-Noble-Noble-Panfilov model ten Tusscher et al. (2004). Future studies may also consider more realistic geometries of the (human) heart Fenton et al. (2005) including fiber orientation Doste et al. (2019) influencing the excitation propagation. Heterogeneities, such as blood vessels and collagen, may not only act as virtual electrodes but also have an impact on the spatio-temporal dynamics Bittihn et al. (2017); Rappel et al. (2022). Modelling this impact in detail requires the use of bidomain models differentiating between intra- and extracellular space. For a more realistic modeling of virtual electrodes, one may use stimuli based on local current injection Feng et al. (2022) and consider perturbation sites of different sizes and shapes where different excitation thresholds due to the strength-extent relation have to be taken into account Bezekci et al. (2015). And again, in all these simulations it has to be checked whether the peaks of high success rate are (still) broad enough to practically apply external shocks precisely within these time frames. Finally, if the findings of the presented study can be confirmed in more detailed simulations of arrhythmic cardiac dynamics and fibrillation, the next challenge will be to predict the peaks in the success rate from measurable observables of the heart.

## Data Availability

The raw data supporting the conclusions of this article will be made available by the authors, without undue reservation.
