# Supplementary material for: The role of pulse timing in cardiac defibrillation
Source: Front Netw Physiol. 2023 Jan 4;2:1007585. doi: 10.3389/fnetp.2022.1007585 (PMC10013017; doi:10.3389/fnetp.2022.1007585)
Supplement: Supplementary file 1 [file DataSheet1.PDF]

frontiers

Supplementary Material

0.1 Calculation of Phase Singularities

For the detection and localization of phase singularities, we first need to define a phase as Iyer and Gray (2001); Bittihn (2015):

$$\theta(\boldsymbol{x}, t) = \mathrm{atan2}(V_{\mathrm{m}}(\boldsymbol{x}, t) - V_{\mathrm{m}}^{\mathrm{ref}}, v(\boldsymbol{x}, t) - v^{\mathrm{ref}}) \in (-\pi, \pi], \tag{S1}$$

where atan2 refers to the two-argument arctangent. Note that $V_{\mathrm{m}}^{\mathrm{ref}} = 0.4\,\mathrm{a.u.}$ and $v^{\mathrm{ref}} = 0.1\,\mathrm{a.u.}$ are reference values, taken from Lilienkamp and Parlitz (2020).
In order to approximate the integral for the phase singularity evaluation, we need to discretize the computational domain, following the method proposed in Iyer and Gray (2001), suggesting an eight pixel integration path:

$$2\pi n_{x,y}^{\mathrm{top}} = \oint_{\mathcal{C}_{x,y}} \nabla\theta\, \mathrm{d}\boldsymbol{l} \approx \sum_{m=2}^{n} \mathrm{diff}(\theta_{x+k_m, y+l_m}, \theta_{x+k_{m-1}, y+l_{m-1}}), \tag{S2}$$

where k_i and l_i parametrize the eight pixel path $\mathcal{C}_{x,y}$ Bittihn (2015); Iyer and Gray (2001):

$$\begin{pmatrix} k_1, \ldots, k_n \\ l_1, \ldots, l_n \end{pmatrix} = \begin{pmatrix} 1 & 1 & 0 & -1 & -1 & -1 & 0 & 1 & 1 \\ 0 & 1 & 1 & 1 & 0 & -1 & -1 & -1 & 0 \end{pmatrix}, \tag{S3}$$

and $\mathrm{diff}(x, y)$ reads:

$$\mathrm{diff}(x, y) := \mod(x - y + \pi, 2\pi) - \pi \in [-\pi, \pi), \tag{S4}$$

The integration path is chosen to enclose a region of $2h \times 2h$, i.e., four grid cells, and hence, four adjacent grid points are considered as possible candidates for the actual position of the spiral tip if the integral yields $n_{\mathrm{top}} = \pm 1$. For simulations, we choose as a condition for a phase singularity to be detected at (x, y) that the topological charge is acceptably close to this value, i.e., $|n_{x,y}^{\mathrm{loc}}| > 0.99$. We follow the convention in Bittihn (2015) and Iyer and Gray (2001) to define the position of the phase singularity to be in the center of this $2h \times 2h$ region.

1 GENERATION OF SPATIOTEMPORALLY CHAOTIC SPIRAL WAVE EXCITATION DYNAMICS

In order to create chaotic transients, we used a variant of the so called cross pacing protocol, see e.g., Lilienkamp and Parlitz (2020). We initialized the computational domain $\mathcal{D}$ with the steady state, $(V_{\mathrm{m}}^*, v^*, w^*) \approx (0, 1, 1)$ and generated a plane wave that travels from the left- to the right-hand side of $\mathcal{D}$. After the wave has passed approximately half of the computational domain, we apply multiple local stimuli on the upper side of $\mathcal{D}$. In order to generate different chaotic states, we varied the time of the pacing onset and frequency of these local stimuli. We let the system evolve for, in total, 1000 ms after generating the very first planar wave in order to ensure that the dynamics is acceptably uncorrelated with the cross pacing initialization. Furthermore, since the chaos is of transient nature, we require each chaotic initial condition

to exhibit spiral waves for at least 2000 ms which guarantees that the chaotic transient does not terminate by itself for a sufficiently long time. This means that for the 10 s long trajectories of chaotic excitation waves, in total 12 s of the trajectories are required to exhibit the spatiotemporal chaos (i.e., to exhibit at least one phase singulartiy). One such generation of a chaotic initial condition is shown exemplarily in Fig. S1. Note that not in all cases, spiral waves are generated with the cross pacing protocol.

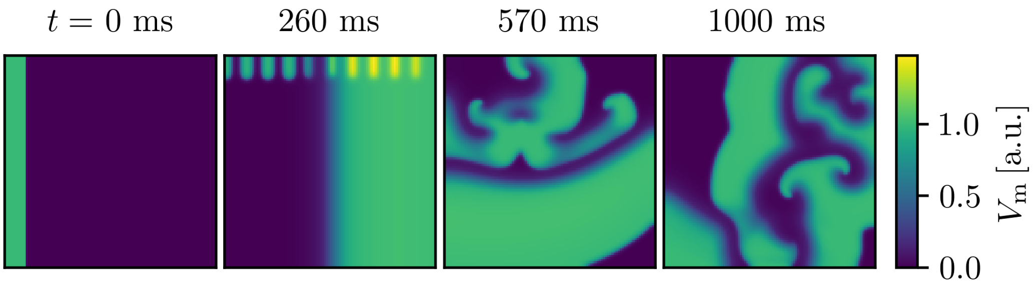

Figure S1. The generation of chaotic initial conditions. A plane wave is initialized on the left-hand side of the computational domain $\mathcal{D}$ and propagates to the right. After the wave passed around half of $\mathcal{D}$, several local stimuli perpendicular (on the upper edge) to the propagation of the initial wave are applied. Time-evolving the system further, spiral waves dominate the domain.

2 CORRELATION COEFFICIENTS OF SUCCESS RATE TIME SERIES

N_pert	250	450	500	550	750	1000
250	1.00**					
450	0.37**	1.00**				
500	0.27**	0.78**	1.00**			
550	0.22**	0.75**	0.85**	1.00**		
750	0.10*	0.51**	0.63**	0.75**	1.00**	
1000	0.03	0.29**	0.36**	0.46**	0.78**	1.00**

Table S1. Spearman correlation coefficients between two time series with N_pert applied, see Fig. 7. Significant results are distinguished by * for $p < 10^{-3}$ and ** for $p < 10^{-7}$.

N_pert	250	450	500	550	750	1000
250	1.00**					
450	0.64**	1.00**				
500	0.55**	0.93**	1.00**			
550	0.45**	0.84**	0.93**	1.00**		
750	0.18**	0.46**	0.58**	0.72**	1.00**	
1000	0.01	0.18**	0.25**	0.35**	0.72**	1.00**

Table S2. Pearson correlation coefficients between two time series with N_pert applied, see Fig. 7. Significant results are distinguished by * for $p < 10^{-3}$ and ** for $p < 10^{-7}$.

$\Delta V_{\mathrm{m}}^{2\times2}$ [a.u.]	0.15	0.30	0.45	0.50	0.55	0.70
0.15	1.00**					
0.30	0.24**	1.00**				
0.45	0.01	0.52**	1.00**			
0.50	0.01	0.49**	0.80**	1.00**		
0.55	-0.02	0.45**	0.80**	0.87**	1.00**	
0.70	-0.09	0.34**	0.69**	0.78**	0.85**	1.00**

Table S3. Spearman correlation coefficients between two time series with $\Delta V_{\mathrm{m}}^{2\times2}$ applied, see Fig. 8. Significant results are distinguished by * for $p < 10^{-3}$ and ** for $p < 10^{-7}$.

$\Delta V_{\mathrm{m}}^{2\times2}$ [a.u.]	0.15	0.30	0.45	0.50	0.55	0.70
0.15	1.00**					
0.30	0.45**	1.00**				
0.45	0.20**	0.76**	1.00**			
0.50	0.17**	0.65**	0.95**	1.00**		
0.55	0.13*	0.56**	0.89**	0.95**	1.00**	
0.70	0.08	0.38**	0.73**	0.82**	0.90**	1.00**

Table S4. Pearson correlation coefficients between two time series with $\Delta V_{\mathrm{m}}^{2\times2}$ applied, see Fig. 8. Significant results are distinguished by * for $p < 10^{-3}$ and ** for $p < 10^{-7}$.

REFERENCES

Bittihn, P. (2015). *Complex Structure and Dynamics of the Heart* (Switzerland: Springer International Publishing), 1 edn.

Iyer, A. and Gray, R. (2001). An Experimentalist's Approach to Accurate Localization of Phase Singularities during Reentry. *Annals of biomedical engineering* **29**, 47–59. doi:10.1114/1.1335538

Lilienkamp, T. and Parlitz, U. (2020). Terminating transient chaos in spatially extended systems. *Chaos: An Interdisciplinary Journal of Nonlinear Science* **30**, 051108. doi:10.1063/5.0011506
